# Science in Story

**Published:** 1875-11

**Authors:** 


Science in Story.—A more ingenious way of beguiling our small boys into a knowledge of how they are made, was never devised than is shown in this most attractive story of Sammie and Sponsie, written by Dr. E. B. Foote, author of Medical Common Sense. Instruction is so skilfully blended with the story that the thread will be lost if there is an attempt to skip the more solid portion of the work, and we do not think any boy can commence the life of the learned little doctor and his mischievous monkey and not follow it to the end. Nor is it attractive alone to children. I doubt if any one would fail to be entertained and instructed by its perusal. The style is so pleasant and funny and the book so full of incident that we can hardly credit the Dr's. assertion in his preface that he never read but one work of fiction in his life. The work is published by the Murray Hill Publishing Company, is well printed on tinted paper, and handsomely illustrated, and will doubtless gladden the heart of many an eager boy the coming Christmas.

---

THE HYACINTH is not only a very beautiful and fragrant flower, but one easily cultivated. This is the season when the bulbs intended for house cultivation should be secured. A word to our lady friends in regard to the best modes of what is known as glass or water culture. First give a preference to the single varieties. They are less beautiful, but are to be preferred to the double ones for the reason that they bloom two or three weeks sooner and are more fragrant. Having selected the bulbs, fill the glasses with pure water, place the bulbs in them, and set them in a dark closet for a fortnight or three weeks, or until the glasses are well filled with fibres. Then expose to the sun; give fresh air occasionally, when the weather will permit. Change the water when it becomes impure. When making the change take out the bulbs and rinse the roots well in clean water; also wash the insides of the glasses thoroughly. The freezing of the water not only endangers the safety of the glasses, but causes the roots to decay. Soft water is always preferable. It should be perfectly clear to show the roots to advantage, and should be on a level with the base of the bulb. Avoid placing the glass within the influence of fire heat. These brief directions, carefully observed, will result in handsome blooms.

